# Supplementary material for: Are carbon emissions trading and green financial instruments synergistic? -Comprehensive quantitative research based on content analysis
Source: PLoS One. 2024 Mar 7;19(3):e0298601. doi: 10.1371/journal.pone.0298601 (PMC10919601; doi:10.1371/journal.pone.0298601)
Supplement: S1 Data — (ZIP) [file pone.0298601.s001.zip › Data/地级市Z_F工作报告.zip!/║╙─╧/╨┬╧τ╩╨/└┤╫╘╣1⁄2╓┌║┼í╛┬φ┐╦╩2╛▌═°í┐_2008.docx]

各位代表：

　　现在，我代表市人民政府向大会作工作报告，请予审议，并请各位政协委员提出意见。

　　一、2007年工作回顾

　　2007年，市人民政府在省委、省政府和市委的领导下，在市人大、市政协监督支持下，团结带领全市人民，深入贯彻落实科学发展观，推动经济转型，构建和谐社会，开拓创新，真抓实干，较好地完成了市十届人大五次会议确定的各项任务。

　　全市发展态势良好。发展质量日益提高。全年实现地区生产总值777.9亿元，比上年增长 （下同）16%；财政一般预算收入41.1亿元、一般预算支出84.2亿元，分别增长28.6%和27.8%，收入和支出三年内均翻了一番；限额以上工业企业实现利润、利税分别增长99.7%和76.1%，均居全省前列。年末金融机构各项存款余额652.6亿元，各项贷款余额461亿元，分别增长5.4%和11.2%。全社会消费品零售总额224.5亿元，增长18.3%；进出口总额13.8亿美元，增长42.8%；其中出口8.6亿美元，增长35.6%。单位生产总值能耗下降5%，完成年度目标。经济后劲不断增强。城镇固定资产投资完成512亿元，增长36.9%，总量稳居全省前列。工业投资完成336亿元，占城镇以上投资比重65.6%。新引进亿元以上项目54个、世界500强企业5家，实际利用外资1.5亿美元，增长15.6%。利用市外资金91.6亿元，增长41%。实施千万元以上项目1450个，比上年增加539个，其中新开工建设项目1078个，增加478个。新飞年产30万台太阳能热水器等48个重点项目开工建设，宝山电厂2×66万千瓦火电机组等46个重点项目竣工投产。民生改善成效明显。省、市两级向群众承诺的20件实事全部完成。城镇居民人均可支配收入11236元、农民人均纯收入4355元，扣除价格因素，分别实际增长13.6%和13%。城镇登记失业率控制在3.3%以内。

　　经济转型步伐加快。一、二、三产业的比例由上年的15.1∶53∶31.9调整为14.9∶53.6∶31.5，分别增长6.1%、20.6%和13.1%。工业发展势头迅猛。限额以上工业增加值增长27.5%，居全省第二位。产业集聚发展态势明显，七大产业集群工业增加值占全市工业增加值的46%。5个省级以上工业园区引进工业企业76家，成为全市新的经济增长点。优势产业支撑作用日益突出，六大主导产业实现利润、利税分别占全市的65.1%和62.1%。重点企业加快发展，主营业务收入超亿元的企业达172户，18户企业分别入选省100户重点企业和50户高成长型企业。新航集团、科隆集团等企业战略重组进展顺利。大力推动直接融资，心连心化肥成功上市，市创业投资公司组建就绪。现代农业水平不断提高。粮食总产74.6亿斤，再创历史新高，优质粮比重达82%。“奶业发展年”活动成效明显，奶牛存栏4.7万头。畜牧业产值突破100亿元，占农业总产值的45%。农业产业化龙头企业和农民专业合作组织快速发展。无公害农畜产品认定、认证工作继续保持全省领先。服务业加速发展。小店综合物流园区基础设施日趋完善。市百货大楼二期工程等一批重点商贸项目竣工投用。新开工商品房361万平方米，竣工179万平方米。旅游综合收入增长29.8%。

　　自主创新得到加强。新增国家级企业技术中心1家、省级企业技术中心5家、省级工程 （技术）研究中心7家。新增省高新技术企业40家、省高新技术产品57项，均居全省首位。市农科院入围全国农业综合科研实力百强院所。新飞电器被认定为国家知识产权企事业示范单位，金龙集团被认定为国家知识产权企事业试点单位。华兰公司被确定为国家级创新型试点企业，“治疗性乙肝疫苗”项目被列入省“十一五”重大科技专项。科隆电器等3家企业被确定为河南省首批知识产权优势企业，数量居全省第一。新增国家名牌产品2个、免检产品6个。专利申请1195件，居全省第三位。高新技术产业增加值占全市工业增加值的27.5%，仍居全省前列。

　　生态建设取得突破。开展水环境综合整治，对7家涉污重点企业和8家化工 （化肥）企业进行深度治理。一次性爆破拆除16台共34.5万千瓦小火电机组，提前三年完成 “十一五”关停任务。水泥立窑生产线全部拆除。县 （市）污水处理厂和垃圾处理场全部建成，市医疗废物处置中心和粪便无害化处理场竣工投用。新增农村沼气用户6万户，沼气入户率达36%，居全省第一。节约集约利用土地， “三项整治”盘活存量土地6360亩，新建成标准厂房49.2万平方米。完成各类造林26.1万亩，凤凰山省级森林公园植树582万株。四条省控河流出境断面水质均达到省定标准，全年空气质量优良级天数达321天。

　　和谐社会建设全面推进。就业再就业工作得到加强。城镇新增就业13.9万人，失业人员实现再就业5.7万人，其中“4050”人员再就业2万人。社会保障体系不断完善。养老金征缴扩面工作稳步推进。新增参保金额1.3亿元、参保人员3.6万人，分别增长16.8%和22.7%。养老金按时足额发放率继续保持100%。11.8万城市低保对象实现动态管理、应保尽保，农村低保人数扩至14万人。新建、改造农村敬老院23所，五保户集中供养率达30.7%。新开工经济适用房17.7万平方米，竣工16.9万平方米；落实廉租住房保障资金2529.2万元，保障住房困难群众3099户。社会事业全面发展。全部免除农村义务教育阶段学杂费，发放城镇低保家庭“两免一补”资金92.2万元，资助学生4860人。完成195所农村中小学校舍改造，配置课桌椅20.7万套，惠及学生41万人。进城务工人员子女入学实现应收尽收。新乡学院挂牌成立，新乡职业技术学院新校区投入使用。乡镇卫生院改造扩建全部完成，改建社区卫生服务中心22个、社区卫生服务站84个，新建、改造标准化村卫生室1020所。成立新乡文化产业投资公司，《新乡市潞简王墓保护条例》通过省人大常委会审议。人口自然增长率控制在6‰以内。食品、药品安全工作不断加强，农产品质量安全检测检验体系初步建立。新闻出版、防震减灾、体育、统计、史志、民族宗教、外事、侨务、档案、气象、测绘、人防等工作都取得了新的成绩。民主法制工作扎实推进。自觉接受市人大及其常委会的监督和市政协的民主监督，严格执行市人大及其常委会通过的决定和决议，人大代表议案、建议和政协委员提案办结率均为100%。加强社会组织建设，基层社会管理体制不断完善。精神文明建设深入开展，成功创建省文明城市。集中开展“百日整治”、争创“四无”单位和“走进矛盾、破解难题”活动，信访总量和赴京去省集体上访量明显下降。加强社会治安综合治理，严厉打击各种违法犯罪活动，社会大局保持稳定，再次荣获全省“平安建设先进市”称号。加快应急体系建设，应对公共危机能力明显提高。创建民族团结进步先进市活动扎实推进。安全生产形势平稳，安全生产事故死亡人数下降21.2%。加强政府自身建设，强化行政监察。大力开展 “行政效率提速年”活动，在全省率先建立全市统一的政府资源招投标平台。强化审计监督，提高财政资金的使用效率。各级政府部门及工作人员的责任、法治和服务意识不断增强，反腐倡廉工作深入开展。

　　城乡统筹发展取得新的成绩。新农村建设稳步推进。全年用于新农村建设的财政支出35.2亿元，比上年增加11.3亿元。连续三年获得省农田水利基本建设 “红旗渠精神杯”。新建、改建农村公路597公里。又解决农村18万人饮水安全问题。60％的示范村实现了村村通自来水、村内道路硬化和垃圾处理规范化。实现村村通光缆、通宽带，通油路的村全部通班车，城乡生活用电同网同价。完成207个自然村“广播电视村村通”工程，新建、改造 “农家店”1010家。新建农村文化大院193个。新型农村合作医疗参合率达96.2%。农村劳动力实现转移就业103.8万人，劳务输出组织化程度不断提高，“长垣厨师”、“长垣防腐”、“新乡海员”入选全国优秀劳务品牌。启动老区三年脱贫计划，完成37个贫困村整村推进脱贫任务，又有3.2万人实现脱贫。农村综合改革深入推进，在全省率先成立惠农信用担保公司，启动实施21个农村集体经济产权制度改革试点。县域经济不断壮大。完成县域生产总值547.8亿元，增长19.2%，总量占全市生产总值的70.4％。辉县市、新乡县、长垣县入选中部县域经济基本竞争力百强县（市）。8县（市）财政一般预算收入全部超亿元。城镇化水平不断提高。新增高速公路通车里程107公里，新建、改造干线公路178公里，被交通部确定为豫北地区唯一的国家公路运输枢纽城市。产业聚集区建设加快，完成基础设施和项目投资97.5亿元，桥北新区建设有了实质性进展。国家卫生城市创建工作取得突破，市区内新建、改造道路10条共12公里；新建、改造提升公厕1200座，旱厕全部消灭；新建垃圾中转站47个；养殖场全部搬迁，彻底改变了人畜混住的局面；新建、改造提升市场25个；整治街巷241条、庭院1633个、“五小”单位4220家；建立了环卫保洁长效机制，居民生活环境明显改观。城镇化率达37.3%，比上年提高1.8个百分点。

　　2007年，我市相继荣获中国城市综合实力百强市、两岸四地城市竞争力百强市、国家园林城市、中国中部最佳投资城市、中国最佳商业城市、国家知识产权试点城市等荣誉称号，连续三届被授予“全国质量兴市先进市”称号，连续四届荣获“全国双拥模范城”称号。

　　各位代表，过去的一年，是我市经济社会又好又快发展、综合实力显著增强的一年，是城乡面貌明显改观、城市形象大幅提升的一年，是人民群众得到实惠较多、生活水平不断提高的一年。成绩来之不易，这是省委、省政府和市委正确领导的结果，是市人大、市政协监督支持的结果，是全市人民同心同德、团结奋斗的结果。在此，我代表市人民政府，向各位人大代表和政协委员，向工作在全市各条战线上的广大工人、农民、知识分子、干部、驻新解放军指战员、武警官兵、公安干警及社会各界人士致以崇高的敬意！向关心、支持新乡发展的朋友们表示衷心的感谢！

　　同时，我们也清醒地看到，我市经济社会发展中还存在不少突出矛盾和问题。一是区域经济整体竞争力不强。全市主要经济指标多数仍处于全省中游。工业主导产业竞争优势不突出，缺乏一批有较强带动作用的大型企业集团和重大工业项目，产业链条延伸不够，创新能力亟待加强。农业基础依然比较薄弱，知名品牌少、附加值低，农民持续增收困难。第三产业发展相对缓慢。二是发展方式尚未根本转变。经济发展与资源、环境的矛盾突出，土地供需异常紧张，单位GDP能耗仍高于全省平均水平。去年曾被省环保局区域限批所有涉水建设项目，后来虽然提前解禁，但节能减排、环境保护特别是卫河流域的水污染防治任务还很艰巨。三是改善民生的力度仍需加大。就业社保、教育卫生、居民住房、食品药品安全等关系群众切身利益的问题依然突出，公共产品和公共服务供给能力有待提高。市场物价上涨较多，低收入群体生活受到较大影响。影响社会稳定的因素仍不同程度存在。城镇建设欠账较多，城乡生活环境仍需大力改善。四是政府自身建设还需进一步加强。政府个别部门和少数工作人员理想信念淡薄、服务意识不强、办事效率不高，还存在虚报浮夸、形式主义甚至滥用职权、违法乱纪等现象，廉政建设仍存在不容忽视的问题。对此，我们将认真对待，切实解决。

　　二、2008年政府工作的主要任务和奋斗目标

　　2008年是贯彻落实党的十七大精神的第一年，是改革开放30周年，我们将面临前所未有的发展机遇，也将面对更加严峻的竞争压力。形势发展要求我们，既要充分把握有利条件，抢抓机遇，乘势而上；又要清醒预见困难矛盾，积极应对，奋力赶超，开创新乡经济社会跨越发展新局面。

　　2008年政府工作的基本思路和主要任务是：认真贯彻党的十七大精神，以科学发展观统领经济社会发展全局，落实宏观调控政策，坚持好字优先、加快发展，积极推进改革开放和自主创新，着力优化经济结构和提高发展质量，继续实施引资项目双带动战略，突出抓好重大工业项目、生态文明村、林业生态、节能减排、城乡建设与管理等五项重点工作，更加重视改善民生和促进社会和谐稳定，推进社会主义经济建设、政治建设、文化建设、社会建设，奋力争先晋位，实现跨越发展。

　　2008年国民经济和社会发展预期目标是：生产总值增长13%，其中，一、二、三产业分别增长5%、15.1%和13%；全社会固定资产投资增长18%，其中工业固定资产投资增长 30%以上；社会消费品零售总额增长15%；地方财政一般预算收入增长15%；城镇居民人均可支配收入增长10%，农民人均纯收入增长8%；城镇登记失业率控制在4%以内；人口自然增长率控制在6.3‰以内；单位生产总值能耗降低5.1%；化学需氧量、二氧化硫排放量分别减少12.4%和24.3%。

　　做好2008年的政府工作，我们必须全面贯彻落实科学发展观，做到五个坚持。坚持改革创新。一切围绕发展，一切服务发展，以改革激发动力，以创新增添活力，注重推进制度建设和创新，努力破解资金、土地、环境容量等发展瓶颈，拓宽发展新思路，谋求发展新举措，切实把经济社会转入科学发展的轨道。坚持提升质量。着力调整经济结构，转变发展方式，推进技术创新，抓好节能减排，实现经济发展、财政增收、群众受益、环境更优。坚持改善民生。从教育医疗、劳动就业、社会保障、群众安居、公共服务等热点难点问题入手，增加财政投入，加大工作力度，使人民群众得到更多的实惠。坚持重点突破。抓事关当前的主要矛盾，抓影响长远的薄弱环节，勇于克难，强力攻坚，以重点突破之功，取推动全局之效，促进经济社会全面协调可持续发展。坚持真抓实干。改进作风，提高效率，营造干事创业的浓厚氛围。超前谋划，求真务实，不事张扬，埋头苦干，充分利用好每一天，认真干好每件事，确保各项工作落到实处。

　　三、推动经济跨越发展

　　以转变经济发展方式和提高经济效益为核心，大力推进经济结构调整，努力实现更大规模、更高水平的发展。

　　（一）坚定不移地实施引资项目双带动战略。加大项目引进力度，带动生产要素集聚，推动产业结构升级，增强经济发展后劲，蓄积发展潜力。提高招商引资的水平。充分发挥全国加工贸易梯度转移重点承接地的优势，围绕“三个500强”投资意向，积极开展产业链招商、以商招商、园区招商和重点项目定向招商。坚持市级领导分包小分队招商制度，各县（市）、区和省级以上工业园区党政正职保证一人常年在外招商。办好比干诞辰3100周年纪念活动，积极参加第三届中部投资贸易博览会和第五届国际投资贸易洽谈会等活动。优化投资环境，提高签约项目合同履约率。确保澳门宝龙城市广场、日本富士电梯、香港佑昌电光源基地等重大项目开工建设。全年新批外商投资企业50家，实际利用外资1.65亿美元，利用市外资金100亿元。抓好重大项目建设。计划实施千万元以上项目830个，总投资1260亿元，年度完成投资455亿元；实施重点项目160个，总投资548亿元，年度完成投资150亿元，其中工业项目87个，总投资190亿元，年度完成投资62亿元。加大华兰系列生物制品、永煤集团中新化工甲醇、宝泉抽水蓄能电站4×30万千瓦机组、南水北调工程新乡段等100个续建项目的投资力度，确保金龙集团年产6000万平方米新型锂离子电池隔膜材料、新航集团年产20万台动力转向器等40个项目竣工投产。开工建设心连心化肥合成氨及复合肥、新乡化纤粘胶短纤维、渠东热电厂2×30万千瓦热电等60个项目。突出抓好我市确定的战略性、产业升级、基础设施和基础产业等四个方面106个重大项目，着力推进原阳桥北河南科技城、宝山二期、宝泉二期、科隆电器年产2万吨高性能电源材料、新亚集团年产20万吨轻量涂布纸、延化化工年产60万吨尿素等重大项目的谋划实施工作。以500千伏和220千伏变电站建设为依托，完善电网格局，为经济发展提供电力支撑。更加注重项目质量。多上市场容量大、产业链条长、科技含量高和符合国家鼓励发展类项目，严格限制高耗能、高排放项目，从源头上把好关，坚决不引不上污染和限制类项目，促进可持续发展。

　　（二）推动产业结构优化升级。进一步扩大产业规模，提升产业层次，促进一、二、三产业全面发展。实施工业跨越工程。抓住国家促进产业集群发展的机遇，大力发展园区经济，增强集群龙头企业的辐射带动能力，延伸产业链条，鼓励中小企业集群发展，力争制冷等七大产业集群销售收入、利税分别增长30%和27%以上。构筑可持续发展的优势产业体系，力争六大主导产业销售收入增长30%以上，利用先进技术改造提升四大传统产业。积极培育优势重点企业，实施市领导分包服务企业制度，着力扶持80户工业优势企业做大做强，打造新乡工业发展的引擎和旗帜；销售收入超亿元的企业达到180户，金龙集团和新飞电器（集团）分别突破160亿元和80亿元。积极引进战略投资者，加快企业战略重组，培育一批跨地区、跨行业、跨所有制的大型企业集团。大力发展现代农业。以发展集约农业、规模农业为目标，以高产、优质、高效、生态、安全农产品为重点，依托龙头企业和农民专业合作组织，大力实施农业品牌战略，提高农业效益，带动农业增产，促进农民增收。切实保护好耕地，努力提高粮食产量。实施小麦高产开发“1346”工程，加快建设优质小麦生产和加工基地、优质小麦种子基地和原阳大米、封丘金银花基地。积极发展高效经济作物；强力推进原阳、延津、长垣、城郊现代农业示范区建设。继续开展好“奶业发展年”活动，新增奶牛存栏2.3万头。做好重大动物疫病防控工作，促进畜牧业健康发展。开展 “农产品质量安全绿色行动”，加大农副产品市场监管力度，做好无公害农畜产品的认定、认证工作。大力实施农业产业化，新培育认定市级重点龙头企业20家以上、农民专业合作社80家以上。加强农田水利基础设施建设，扩大引黄灌溉面积，扎实做好各项防汛抗灾准备工作。加快现代服务业发展。以发展生产性服务业为重点，着力提升服务业发展整体水平。加快小店物流港、新乡粮食物流园区、原阳农产品贸易中心和新钢钢材物流园等项目建设，积极融入中原城市群物流核心圈。加快银行、证券和保险业发展，扩大投资基金、债券、信托融资规模；加快企业上市步伐，力争5家企业实现上市或再融资；强化信用建设，保持信贷均衡有效投入。加快发展商贸服务业，确保沃尔玛、华彬阳光广场等项目竣工投用，努力形成特色商业街、商业步行街和重点商贸项目共同支撑的商业发展格局。以南太行、牧野古文化、沿黄生态三大旅游带为主线，加快以道路为重点的基础设施建设，高起点规划和开发八里沟等重点景区，强力推进百泉景区复兴工程。

　　（三）强化自主创新能力。充分发挥科教优势，推动经济发展由要素驱动型向创新驱动型转变。高度重视人才。全面落实政策措施，健全激励机制，防止人才流失，引进高层次人才，充分调动各类人才的积极性、主动性和创造性。加快产学研结合。继续加大技术创新投入力度，优化整合创新资源，完善创新服务体系，引导企业与高等院校、科研单位进行多层次研发合作，加快科研成果的产业化进程，实现科研创新与经济发展的有效对接。加强农业科技创新，推进农业科技创新中心建设，促进农业科技成果转化与推广。增强企业创新能力。积极引导有条件的企业组建研发中心，新增市级工程（技术）研究中心25家、省级工程（技术）研究中心5家。发展壮大高新技术产业，促进高新技术产业聚集发展，争创国家级、省级产业基地，重点组织申报国家火炬计划3项、省级高新技术产业化项目3项、省企业自主创新项目3项，新增省高新技术企业20家、省高新技术产品25项，高新技术产业增加值占全市工业增加值的比重达到28%。申报省级以上科技计划项目40项，重点在新材料、新能源、节能环保、生物医药、电子信息等方面取得突破。组织实施好 “硅片材料加工数字化装备及关键技术”、“治疗性乙肝疫苗”等重大科技项目，着力突破制约经济发展的核心技术和关键技术。大力实施名牌战略。引导企业重视品牌建设，形成品牌密集的优势行业。争创中国名牌产品2个、省名牌产品10个，国家免检产品5个、省免检产品15个。强化专利工作。加强知识产权的开发、应用和保护，全市专利申请量保持在1000件以上，发明专利比例力争达到20%。

　　（四）深入推进改革开放。不断完善科学发展的体制机制，提高经济发展外向度，增强经济发展动力。加大改革攻坚力度。进一步深化国企改革，基本完成市属国企改制工作，积极推进局委属企业和集体企业改革，完成商贸流通企业改制工作。帮助已改制企业解决好遗留问题，鼓励引进战略合作伙伴，推动国有资产向优势行业集中。推进市直事业单位分类改革，扎实做好事业单位登记管理工作。大力发展非公有制经济，加强信用担保、社会化服务等体系建设，为民营经济发展创造良好环境。做好统一内外资企业所得税工作。加强政府投资项目管理，完善国有资产管理体制，确保国有资产保值增值。加快市商业银行上市步伐，深化农村信用社改革。提高对外开放水平。加快新乡口岸建设和大通关建设，申请设立新乡海关和出入境检验检疫机构。继续壮大出口队伍，力争新增进出口经营权登记企业310家，进出口总额完成16亿美元，其中出口10亿美元。优化出口商品结构，提升高新技术产品、机电产品出口比重。扩大先进技术、重要资源及原材料进口。鼓励有条件的企业扩大境外投资，开展国际化经营。完成对外承包工程和劳务合作营业额850万美元，外派国（境）外劳务人员1100人次。海员园区建成投用。

　　（五）加快城乡一体化步伐。以“三位一体”系统工程为载体，以“四个城市化”为目标 （即农民居住环境、就业结构、公共服务和消费方式城市化），以推进“六个一体化”为主要内容 （即城乡发展规划、产业布局、基础设施、公共服务、劳动就业和社会管理一体化），促进城乡融合互动，统筹城乡发展。建设社会主义新农村。以改善人居环境、提高农民生活质量为突破口，坚持“抓好示范村、建设中心村、控制一般村”的原则，分类指导，全力推进以新型农村住宅社区建设为标志的生态文明村建设，确保1361个村达到生态文明村阶段性建设标准。抓好“一县一镇百村”工程，加快重点区域村庄的整治整合，推进重点区域以外的中心村建设。加快城乡基础设施建设步伐，改造县乡公路200公里、通村公路600公里，再解决17万农村人口的饮水安全问题，新增沼气用户3万户，完成60个以上自然村“村村通电话”工程和206个自然村“广播电视村村通”工程，每个县（市）建立一个自动气象站。落实好粮食直补、农业生产资料综合直补、良种补贴、农机购置补贴等各项惠农补贴。继续推进老区三年脱贫计划，完成50个贫困村整村推进脱贫任务，确保再有3.17万农村贫困人口脱贫。发展壮大县域经济。围绕“富民强县”的目标，强化城市意识、工业意识和开放意识，强化重大项目的谋划与建设，大力发展民营经济、园区经济、产业集群和特色产业，壮大经济实力，提高发展水平。加大对困难地区的扶持力度，促进县域经济均衡发展。力争完成县域生产总值594亿元，增长14%。推进新乡都市区建设。各级产业聚集区实施基础设施建设项目216个，完成投资24.5亿元。引导企业入驻各级产业聚集区，使其成为全市对外开放和招商引资的战略平台，成为承接资本、技术、人才和产业转移的核心区域。推进国家级、省级工业园区的申报工作，争取桥北新区等一批产业聚集区获得省批准。完善新乡都市区交通网络，开工建设新晋高速、郑新黄河大桥连接线、济东高速黄河大桥、京珠高速安新段扩建等工程，新改建安信公路新乡段等7条共295公里的干线公路。推进新区、新乡高新技术产业开发区、凤泉区、新乡工业园区一体化，促进市区东部隆起。做好新乡与郑州双核城市规划编制工作，推进新郑产业带建设，加快建设桥北新区，构筑新乡与郑州城市功能和产业对接支点。吸引人口向城镇集聚，全市城镇化率提高1.7个百分点。

　　四、加快推进生态文明建设

　　牢固树立生态文明理念，将环境保护和节能减排作为转变经济发展方式的突破口，严格实行节能减排问责制和“一票否决”制，努力建设生态新乡。

　　（一）加强林业生态建设。抓住林业生态省建设机遇，围绕“一山、一带、三环、三网、三通道”，做好生态绿化工作，完成各类造林31.8万亩，林木覆盖率达到25%以上，确保国家森林城市创建成功。加快林业生态市建设步伐，长垣、原阳、辉县年内力争建成省级林业生态县，全市争取率先在全省建成林业生态市。加快凤凰山省级森林公园建设，完成植树造林606万株，确保三年建设任务大头落地。大力发展林业产业，全面完成集体林权制度改革任务。

　　（二）加大污染治理力度。严格执行“三次超标处理”新机制，对违法排污企业进行停产治理、限期治理直至依法关闭，强化卫河治理，全面提高域内河流水质。开工建设新亚集团10万吨白水回用及污水深度处理综合利用项目，推进建设新乡县二次污水处理厂和新乡工业园区污水处理工程。加强污水、垃圾处理费征收工作，确保各县（市）污水处理厂和垃圾处理场正常运行，市区污水处理厂全部加装脱氮装置。燃煤机组全部建成脱硫设施并达标稳定运行。

　　（三）强化能源资源节约。健全落后产能退出机制，淘汰小化工、小水泥、小石渣等落后生产能力。加强重点领域、行业和企业的节能降耗工作，加快推进孟电集团、天瑞水泥和平原同力水泥生产线低温余热电站项目，干法水泥生产线全部实现余热发电。开展大型公共建筑和政府机关办公楼节能改造示范工程。上半年县以上城市全面禁止使用实心黏土砖。继续开展土地 “三项整治”，加强对闲置和低效土地的利用。加大标准厂房建设力度，新开工标准厂房142万平方米，竣工107万平方米。

　　（四）大力发展循环经济。编制《新乡市循环经济发展规划》，推进生物质能（秸秆）发电和太阳能开发利用。抓好心连心化肥合成氨生产能量系统优化、天丰钢板新型节能墙体材料、天意新能源太阳能路灯等重点节能项目。推进新乡纸制品工业园、黄河畜牧有限公司、河南恒友牧业发展有限公司等省级循环经济试点建设，争取赵固循环经济工业园纳入省级循环经济试点。

　　五、推动城市建设再上新台阶

以“新城杯”竞赛活动为载体，立足城市存量资源挖潜，拓展发展空间，完善城市功能，推进城市内涵式发展，促进城镇建设，改善人居环境。

　　（一）着力提升城市品位。坚持旧城改造与新区建设并重，按照“政府主导、统一规划、市场运作、群众自愿”的原则，积极吸引社会资金参与旧城、“城中村”改造和新区建设，推进集中连片开发，提高集约化建设水平。全面启动主次干道两侧42个“城中村”综合开发工作，完成段村、留庄营改造拆迁任务。关注京广铁路以西区域的发展。将旧城改造和房地产开发相结合，推动房地产业加快发展，新开工商品房100万平方米，竣工80万平方米。加大火车站广场等十大特色建筑群建设力度，抓好新乡大剧院等新区标志性工程，努力提升城市形象。

　　（二）不断完善公共服务功能。强化城市基础设施建设，加快市域路网体系建设，贯通大外环，完成4条共10公里的城市道路建设任务。完善城市污水处理配套管网，建成污水管道8.7公里。铺设城市集中供热管网20.7公里，市区新增供热面积200万平方米。开工建设汽车客运东站和货运东站。加快新区公共服务设施建设，市政公园基本建成。完成牧野湖水系景观建设，加快推进西孟姜女河和卫源湖生态景观配套工程，确保卫河市区段下游截污工程和赵定排蓄水配套工程竣工。

　　（三）全面提高城乡建设管理水平。坚持高起点规划，抓好城市总体规划修编工作。实行建设规划行政首长负责制，建立城乡一体的规划管理和执法体系，严查各类违法建设行为，确保城市规划审批执行率100%。深入开展“新城杯”竞赛活动，通过市场化运作，多渠道筹措资金、高标准建设，推进县城及重点镇改造，吸引人口和生产要素聚集，提升城镇形象，使其成为县域经济新的投资增长点。大力开展城乡环境综合整治，确保8条快速路沿线村庄和城区主次干道等区域的环境整治取得明显效果，完成新中大道绿化任务。持续开展爱国卫生运动，巩固国家卫生城市创建成果，按照“全覆盖、无缝隙”的要求，坚持以创促建、促改，完善城市保洁、“五小”治理等长效机制，逐步实现城市精细化和数字化管理，使市区面貌明显改观，建设宜居城市。

　　六、加快以改善民生为重点的社会建设

　　坚持以人为本，建设和谐新乡，着力改善和保障民生，发展社会事业，办好省、市承诺的20件实事，使广大群众学有所教、劳有所得、病有所医、老有所养、住有所居，得到更多实惠。

　　（一）大力发展教育卫生事业。把教育置于优先和突出位置，强化教育资源整合，优化教育资源配置，着力促进教育均衡发展。努力改善农村办学条件，加强师资队伍建设。提高农村义务教育阶段中小学公用经费标准和贫困家庭寄宿生生活费补助标准。做好义务教育阶段“两免一补”工作，从春季起，免费为农村义务教育阶段的所有学生提供教科书；从秋季起，免除城市义务教育杂费。完成170所共9万平方米的农村中小学校舍改造。保证进城务工人员子女入学应入尽入，同城同待遇。继续做好高等院校、高等职业学校和中等职业学校家庭经济困难学生的资助工作。推进新乡一中新校区和新区中学建设，提高高中阶段教育普及率。进一步做好为驻新高校服务工作，支持新乡学院和新乡职业技术学院加快发展，打造高素质人力资源培育基地。努力解决群众看病难、看病贵问题，新型农村合作医疗补助标准由40元提高到80元。为103个乡镇卫生院配置设备，完成生态文明村标准化村卫生室改造。在全市二级以上公立综合医院推行惠民病房或病床，单病种最高限价由88种扩大到100种。加大对各级各类医疗服务机构的监管，全面提高医疗服务管理水平，建立和谐医患关系。完善重大疾病防控体系，提高公共卫生事件应急处理能力。

　　（二）扎实做好就业和社会保障工作。千方百计扩大就业，90%以上的乡镇建立劳动力市场，形成城乡一体的公共就业信息网络。落实支持自主创业和自谋职业的各项政策，确保城镇新增就业7万人，下岗失业人员再就业2.4万人，其中“4050”人员再就业8800人。完成再就业培训2.5万人，发放再就业小额贷款6000万元。抓好26万农村劳动力转移引导性培训和职业技能培训，转移就业105万人。继续做好零就业家庭就业援助工作，使市区所有零就业家庭实现至少一人稳定就业。认真贯彻《劳动合同法》，确保各类企业与劳动者依法签订劳动合同，构建和谐劳动关系。实施职工带薪休假制度，建立职工工资正常增长和支付保障机制。不断完善社会保障体系，新增养老保险参保职工3万人，提高企业退休人员养老金水平。新建、扩建40所农村敬老院，五保户集中供养率达到40%。全面开展城镇居民医疗保险试点工作。推进失业保险市级统筹。完善城乡社会救助体系。城市低保补差标准提高到95元，农村低保标准不低于40元。市区新开工经济适用房20万平方米，竣工15万平方米，新增廉租住房1.5万平方米。鼓励社会力量兴办福利事业，推进新乡市惠民院等项目建设。

　　（三）大力发展文化事业和其他社会事业。弘扬“厚善、崇文、敬业、图强”的城市精神，广泛开展形式多样的文明创建活动，提高全社会文明程度。大力发展社区文化、广场文化、企业文化、校园文化、节庆文化和民间文化，完成生态文明村文化大院建设任务，广泛开展群众文化活动，健全公共文化服务体系，繁荣城乡文化事业。深入开展全市非物质文化遗产普查和保护工作，继续做好潞简王墓世界文化遗产申报工作。加快平原博物院、“三合一”项目（青少年活动中心、科技馆、妇女活动中心）等一批公益文化项目建设步伐。着力发展文化旅游业、印刷包装业和工艺品制作业，形成新乡文化产业特色。完成文化事业单位转企改制，培育文化产业骨干企业，新乡文化产业投资公司规范运营。稳定低生育水平，优化人口结构，提高人口素质。积极开展群众体育活动，举办新乡市第九届运动会。依法保护妇女、未成年人、残疾人和老龄人的合法权益。落实好老干部的“两个待遇”。大力支持国防建设，做好优抚和退伍安置工作，启动全国双拥模范城“五连冠”创建工作。认真做好新闻出版、广播电视、防震减灾、审计、统计、史志、外事、侨务、档案、气象、测绘、人防等工作。

　　（四）切实加强社会管理工作。创新社会管理体制，提高社会管理水平。强化城乡社区服务功能，创建和谐社区。组织好第六届村委会换届选举工作。完善公共安全应急机制，提高应急管理水平。开展产品质量和食品安全专项整治，扩大农产品市场准入范围。加强市场价格监管，打击价格违法行为，落实好物价补贴工作。推进信访工作制度建设，构建适应形势发展的群众工作网络，健全重大事项信访评估制度，有效预防和化解各类社会矛盾。严格落实安全生产责任制，加强管理监督，坚决遏制重特大事故发生。积极开展普法教育，引导全社会自觉学法用法守法。加强社会治安综合治理，防范和打击各类违法犯罪行为，深化“平安新乡”创建活动。健全维护民族团结的长效机制，加快少数民族聚居地区经济社会发展。依法管理宗教事务，发挥宗教界人士和信教群众在促进经济社会发展中的积极作用。发挥工会、共青团、妇联等人民团体桥梁纽带作用，支持人民团体积极参与社会管理和公共服务。

　　七、切实加强政府自身建设

　　大力推进政府管理创新，转变政府职能，提高行政效能，强化政风建设，努力建设服务型政府。

　　（一）规范行政行为。加强民主法制建设，自觉接受市人大及其常委会的法律监督、工作监督和市政协的民主监督，进一步提高人大代表议案、建议和政协委员提案的办理质量，主动接受社会各方面的监督。扩大人民民主，拓宽民主渠道，建立健全公众参与、专家论证和政府决定相结合的行政决策机制，推进依法、科学、民主决策。坚持依法行政，严格按照法定权限和程序办事，规范权力运行。落实行政执法责任制，完善行政问责制和政府绩效考核体系，畅通行政复议渠道，提高执法质量，努力做到有权必有责、用权受监督、侵权要赔偿、违法要追究，促进社会公平正义。提高公务员管理的法制化、规范化水平，建设高素质的公务员队伍。

　　（二）提高服务水平。根据上级部署，积极稳妥地推进行政管理体制改革，进一步转变政府职能、理顺关系、优化结构、提高效能。加强和改善经济调节、市场监管的同时，更加注重社会管理和公共服务，改进公共服务方式，优化公共资源配置，加大对“三农”、社会保障、医疗卫生、教育、节能减排等领域的投入，建设惠及全民的基本公共服务体系。加强各级行政服务中心建设，推进政府部门行政审批职能整建制进中心工作，健全联审会办流水线运行机制，简化程序，压缩时限，提高质量，巩固完善行政效率提速长效机制。认真组织实施《国务院信息公开条例》，加快电子政务建设，完善政务公开、办事公开、新闻发布等制度，提高政府工作透明度，使人民群众更有效地监督政府。

　　（三）转变工作作风。始终牢记宗旨，增强公仆意识，高度关注群众的安危冷暖，主动解决各种热点难点问题，尽最大努力为群众办好事、办实事。坚持以促进新乡发展为己任，树立忧患意识、危机意识，强化机遇观念、竞争观念，加强学习，勇于创新，着力扫除影响和制约发展的各种障碍。保持奋发有为的精神状态，克服不思进取、推诿扯皮的不良习气，把精力用到干事业抓发展上，一抓到底、务求实效。弘扬真抓实干、雷厉风行的工作作风，深入基层、深入实际，敢于触及矛盾，勇于承担责任，善于创造性地开展工作。大力精简会议、文件，严格控制检查、达标、评比活动，力戒形式主义和官僚主义。

　　（四）加强廉政建设。把反腐倡廉放在更加突出的位置，旗帜鲜明地反对腐败。加强制度建设，真正做到用制度管权、按制度办事、靠制度管人，扎实推进惩治和预防腐败体系建设。积极发挥监察、审计等部门的作用，加强对惠农、惠民资金使用的监管，确保及时足额发放。发挥《行风热线》等舆论监督的作用，扩大社会监督。充分运用机关科室（机构）季评结果，重点加强对领导干部、重要部门和关键岗位的行政执法监督，让权力在阳光下运行。坚决纠风治乱，强化专项治理，狠刹损害群众利益的各种不正之风，着力优化经济发展环境。严肃查办案件，对于各类违纪违法案件特别是贪污贿赂、权钱交易等案件，要一查到底；对于腐败分子，依法严惩，绝不姑息手软。

　　各位代表！新乡的发展已经步入新时期，展望未来，我们既满怀信心，又深感责任重大。让我们高举中国特色社会主义伟大旗帜，深入落实科学发展观，开拓进取，扎实工作，为夺取全面建设小康社会新胜利、早日建成中原城市群强市而努力奋斗！
